# Assessment of cerebral blood flow with magnetic resonance imaging in children with sickle cell disease: A quantitative comparison with transcranial Doppler ultrasonography

**DOI:** 10.1002/brb3.811

**Published:** 2017-10-14

**Authors:** Paula L. Croal, Jackie Leung, Przemyslaw Kosinski, Manohar Shroff, Isaac Odame, Andrea Kassner

**Affiliations:** ^1^ Physiology & Experimental Medicine The Hospital for Sick Children Toronto ON Canada; ^2^ Institute of Medical Science University of Toronto Toronto ON Canada; ^3^ Department of Diagnostic Imaging The Hospital for Sick Children Toronto ON Canada; ^4^ Division of Haematology/Oncology The Hospital for Sick Children Toronto ON Canada

**Keywords:** cerebral blood flow, magnetic resonance imaging, pediatric, sickle cell disease, TCD

## Abstract

**Introduction:**

Transcranial Doppler ultrasonography (TCD) is a clinical tool for stratifying ischemic stroke risk by identifying abnormal elevations in blood flow velocity (BFV) in the middle cerebral artery (MCA). However, TCD is not effective at screening for subtle neurologic injury such as silent cerebral infarcts. To better understand this disparity, we compared TCD measures of BFV with tissue‐level cerebral blood flow (CBF) using arterial spin‐labeling MRI in children with and without sickle cell disease, and correlated these measurements against clinical hematologic measures of disease severity.

**Methods:**

TCD and MRI assessment were performed in 13 pediatric sickle cell disease patients and eight age‐matched controls. Using MRI measures of MCA diameter and territory weight, TCD measures of BFV in the MCA [cm/s] were converted into units of CBF [ml min^−1^100 g^−1^] for comparison.

**Results:**

There was no significant association between TCD measures of BFV in the MCA and corresponding MRI measures of CBF in patients (*r *=* *.28, *p *=* *.39) or controls (*r *=* *.10, *p *=* *.81). After conversion from BFV into units of CBF, a strong association was observed between TCD and MRI measures (*r *=* *.67, *p *=* *.017 in patients, *r *=* *.86, *p *=* *.006 in controls). While BFV in the MCA showed a lack of correlation with arterial oxygen content, an inverse association was observed for CBF measurements.

**Conclusions:**

This study demonstrates that BFV in the MCA cannot be used as a surrogate marker for tissue‐level CBF in children with sickle cell disease. Therefore, TCD alone may not be sufficient for understanding and predicting subtle pathophysiology in this population, highlighting the potential clinical value of tissue‐level CBF.

## 1 | INTRODUCTION

Sickle cell disease (SCD) is the most commonly occurring monogenic hemoglobinopathy and is characterized by microvascular occlusion and hemolytic anemia. It is associated with devastating neurologic injury at a young age, as children with SCD are at high risk of both overt ischemic stroke and silent cerebral infarcts (SCI). SCI are defined as focal white matter hyperintensities on $T_2$-weighted magnetic resonance imaging (MRI) in the absence of clinical deficits, and occur in approximately 40% of children with SCD by the age of 18 (Bernaudin et al., 2011; DeBaun, Sarnaik, et al., 2012; Miller et al., 2001; Pegelow et al., 2002). The presence of SCI in children have been shown to be associated with cognitive decline, poor school performance, and pose a significant risk factor for both future overt ischemic stroke and further SCI (Miller et al., 2001; Schatz, Brown, Pascual, Hsu, & DeBaun, 2001). Therefore, it is important to reliably identify pediatric SCD patients at greatest risk of ischemic injury through the development of more robust tools for assessing cerebral physiology.

The risk of overt ischemic stroke in children with SCD is currently stratified according to transcranial Doppler ultrasonography (TCD) measurements of blood flow velocities in the middle cerebral artery ($BFV_{MCA}$) (Adams, McKie, Hsu, et al., 1998). Based on the clinically recommended $BFV_{MCA}$ threshold of 200 cm/s, above which children with SCD will be prescribed chronic transfusion therapy, the relative risk of primary overt stroke has declined by over 90%. However, it is worth noting that despite high sensitivity to overt stroke, TCD screening suffers from low specificity, correctly predicting stroke only 36% of the time (Adams et al., 1997), potentially leading to transfusion therapy in cases that are not medically necessary. This can result in an unnecessary burden placed on both the patient and the healthcare system.

Although TCD screening has been successful for prevention of overt strokes in children with SCD, TCD has proven ineffective at screening for more subtle injuries such as SCI (Arkuszewski et al., 2011; Bernaudin et al., 2015). To date, there is no established association between TCD and SCI (DeBaun, Armstrong, et al., 2012), illustrated by the high cumulative risk for SCI observed in patients without abnormal TCD. Furthermore, results from the Silent Cerebral Infarct Transfusion Multi-Center Clinical Trial demonstrated that 14% of patients not on treatment but with SCI suffered from further ischemic damage despite TCD velocities below the transfusion threshold. This recurrence rate was significantly reduced in the transfused group, but ischemic endpoints were still observed in 6% of patients (DeBaun et al., 2014).

The etiology of SCI is complex and likely multifactorial (Debaun, Derdeyn, & McKinstry, 2006; Dowling, Quinn, Rogers, & Buchanan, 2010), but recent research has proposed a strong association with tissue-level hypoperfusion (Bernaudin et al., 2015). As such, the lack of relationship between SCI and TCD measurements potentially arises from an inability to detect changes in tissue-level cerebral blood flow (CBF) using TCD measures of $BFV_{MCA}$. Therefore, in order to gain insight into why TCD is not effective in predicting SCI, a better understanding of how BFV obtained in a major artery relates to microvascular CBF is needed. $BFV_{MCA}$ has been assumed to be a surrogate marker for CBF in healthy adults (Battisti-Charbonney, Fisher, & Duffin, 2011), but recent studies in children with SCD reported no significant correlation between TCD measurements of $BFV_{MCA}$ and MRI measurements of tissue-level CBF (Helton et al., 2009; Strouse et al., 2006). However, as these studies did not provide pediatric control data, it is difficult to interpret whether this lack of agreement reflects underlying pathophysiology, technical discrepancies or both. Firstly, small vessel pathology in SCD may not necessarily manifest as abnormal bulk flow in large cerebral arteries (Hartmann et al., 1991). Secondly, TCD measurements of BFV only reflect CBF if both the cross-sectional area of the vessel and tissue weight of the corresponding region supplied by the vessel remain constant across individuals (Schatlo & Pluta, 2007). These factors were not accounted for in previous studies.

The purpose of this study was twofold. We first compared TCD measures of $BFV_{MCA}$ and MRI measures of tissue-level CBF in healthy controls and children with SCD. This was followed by a second comparison after converting the BFV measures into units of CBF. By doing so, we aimed to establish whether converting TCD values into comparable units will more accurately portray tissue-level physiology and pathophysiology. We further validated these measurements against arterial oxygen content ($CaO_2$) derived from hemoglobin and oxygen saturation, which are hematologic factors associated with ischemic risk (DeBaun, Sarnaik, et al., 2012; Prohovnik, Hurlet-Jensen, Adams, De Vivo, & Pavlakis, 2009; Quinn, Variste, & Dowling, 2009).

## 2 | MATERIALS AND METHODS

### 2.1 | Patients

Retrospective data from 13 children with documented SCD (genotype HbSS, ages 11–18 years) were included in this study. The patients were recruited from the hematology clinic at our institution as part of a larger prospective study. Patients with a history of focal neurologic events lasting more than 24 hr, history of blood transfusion therapy (chronic, emergency, and/or preoperative), or hospital admission within 3 months prior to recruitment were excluded. In addition, data from eight healthy age-matched children (ages 11–17 years) with no history of cerebrovascular disease were included as controls. All procedures described in this study were approved by our Institutional Research Ethics Board. Informed written consent was obtained from each participant or parent/guardian.

### 2.2 | TCD protocol

Blood flow velocities of the right and left middle cerebral artery (MCA) of all subjects were obtained by an experienced ultrasound technician (A.M.) using a commercial duplex ultrasound system (iU-22 xMatrix; Philips Electronics, Best, the Netherlands) equipped with a S5-1 2.0 MHz transducer. With the subject in supine position, the ultrasound probe was placed on the acoustic temporal window to locate the M1 segment of the MCA. The time-averaged mean maximum velocity (TAMMV) for each hemisphere was recorded at least three consecutive times and the maximum TAMMV used as a measure of $BFV_{MCA}$.

#### 2.3 | MRI protocol

MRI was performed on a 3.0T clinical MRI scanner (MAGNETOM Trio, Siemens Medical Solutions, Erlangen, Germany) using a 32-channel head coil. For tissue-level CBF measures, pulsed arterial spin-labeling (ASL) data were acquired using a clinically available sequence (2D-EPI PICORE Q2TIPS: $TI_1$ = 700 ms, $TI_2$ = 1800 ms) (Luh, Wong, Bandettini, & Hyde, 1999). A total of 91 dynamics were acquired, which consisted of an $M_0$ calibration image followed by 45 tag-control image pairs that were used to isolate the blood signal. To detect the presence of stenosis within intracranial vessels and measure MCA diameter, 3D time-of-flight magnetic resonance angiography (MRA) images were acquired. To identify the presence of silent infarctions and other white matter hyperintensities, $T_2$-weighted fluid attenuated inversion recovery (FLAIR) images were acquired. For estimation of tissue volume of the MCA perfusion territories, high-resolution $T_1$-weighted anatomical images were acquired using a 3D MPRAGE sequence. Specific scan parameters are provided in Table 1.

#### 2.4 | MRI analysis

##### 2.4.1 | Assessment of structural MRI

Anatomical MRI data (MPRAGE, FLAIR, MRA) were reviewed by an experienced neuroradiologist (M.S.) to screen for presence of SCI or vessel stenoses. Silent infarctions were defined as focal areas (<3 mm) of high intensity in the cortical and subcortical regions on FLAIR images. Cerebral vasculopathy was scored based on focal or diffuse changes in the caliber of intracranial arteries observed on the MRA.

##### 2.4.2 | Calculating cerebral blood flow from ASL ($CBF_{ASL}$)

ASL data were analyzed in MATLAB (Mathworks Inc.). Raw data underwent motion correction, Gaussian spatial smoothing (FWHM 5 mm), and coregistration to the subject's $T_1$-weighted anatomical image. A CBF-weighted map was calculated by subtraction of tag and control images ($\Delta M$), and converted into absolute CBF by fitting to a single-compartment kinetic model on a voxel-wise basis (Wong, Buxton, & Frank, 1998a):

$$CBF_{ASL} = \frac{\lambda \cdot \Delta M}{2 \cdot \alpha \cdot M_0 \cdot TI_1 \cdot e^{\frac{TI_2}{T_{1a}}}} \quad (1)$$

where $\lambda$ is the blood/tissue water partition coefficient (assumed 0.98 ml/g) and $\alpha$ is the inversion efficiency (assumed 95%). Assumed values of $T_{1a}$ were taken as 1818 ms for SCD patients (Václavů et al., 2016) and 1650 ms for healthy controls (Alsop et al., 2015).

Mean CBF within the grey matter of the MCA territories was calculated for each subject. This was performed by defining a left and right MCA perfusion territory mask on the MNI 152 1 $mm^3$ resolution standard-space $T_1$-weighted structural template image (Montreal Neurological Institute, Canada). The regions were manually drawn slice by slice on the $T_1$-weighted image using established atlas definitions as a reference (Moeller & Reif, 2013). The mask was then nonlinearly coregistered to each subject's $T_1$-weighted anatomical image (FNIRT, FSL). A second mask defining the grey matter regions of the brain was segmented from the $T_1$-weighted anatomical images for each subject (FSL-FAST) (Zhang, Brady, & Smith, 2001). The masks were combined so that regional mean CBF could be computed in the grey matter of the defined left and right MCA territories.

##### 2.4.3 | Calculating cerebral blood flow from TCD ($CBF_{TCD}$)

TCD measurements of $BFV_{MCA}$ [cm/s] were converted into units of CBF [ml/100 g/min] to determine if it would improve its correlation with tissue-level ASL data. The conversion involved calculating the bulk flow through the vessel and the distribution of the blood into the tissue by factoring in the cross-sectional area of the MCA ($A$) and the overall weight of the perfusion territories supplied by the MCA ($T$) (Schatlo & Pluta, 2007). Assuming a laminar flow profile, the mean velocity across the vessel lumen is half the peak BFV, and the relation was expressed as:

$$CBF_{TCD} = \frac{BFV_{MCA}}{2} \times \frac{A}{T} \quad (2)$$

The physiological parameters $A$ and $T$ were interpreted from the MRI data of each patient. In brief, using in-house software (MATLAB, Mathworks Inc.), the M1 portion of each MCA was cropped from the MRA image and automatically segmented into vessel wall contours by applying an image-based threshold algorithm (Figure 1). The mean diameter between vessel walls was estimated to derive the cross-sectional area. Brain tissue volume in the right and left MCA territories was determined by summing all respective voxels designated by the

**TABLE 1** MRI sequence parameters

| Sequence | TR (ms) | TE (ms) | FOV (mm) | Voxel size ($mm^3$) | Duration (min:s) |
|---|---|---|---|---|---|
| Pulsed ASL | 2,500 | 13 | 220 | 3.4 × 3.4 × 4.5 | 3:52 |
| 3D time-of-flight MRA | 20 | 3.59 | 200 | 0.5 × 0.5 × 0.5 | 5:18 |
| $T_2$-weighted FLAIR | 9,000 | 85 | 220 | 0.8 × 0.7 × 4.5 | 4:32 |
| $T_1$-weighed 3D MPRAGE | 2,300 | 2.96 | 256 | 1.0 × 1.0 × 1.0 | 5:03 |

TR, repetition time; TE, echo time; FOV, field of view.

MCA mask. Subsequently, an estimation of weight of the MCA territory can be acquired by converting the MRI-derived volumes of parenchyma into grams per milliliter based on previously measured brain tissue density (Frontera, 1958; Stephan, 1960). The resulting measure, $CBF_{TCD}$, represents the rate of blood flow in the MCA distributed across the brain tissue of the corresponding MCA territory.

#### 2.4.4 | Hematologic variables

Hematologic values were recorded for SCD patients from complete blood count reports on blood samples obtained during routine phlebotomy at the institution's sickle cell clinic. Daytime $SpO_2$ was measured using a pulse oximeter (Invivo Expression, Gainsville, USA).

#### 2.4.5 | Statistical analysis

Statistical analysis was performed using *R* (R Core Team, 2011). Data were initially tested for normality using the Shapiro–Wilk test combined with visual inspection of Q–Q plots to determine whether parametric or nonparametric descriptors and statistical tests were appropriate. Between-group differences were assessed using a Welch's *t* test for normally distributed data, and Wilcoxon Rank Sum test for non-normally distributed data. Correlations between BFV and CBF measurements (averaged across right and left hemispheres) were assessed using either the Pearson correlation coefficient ($r$) or the nonparametric equivalent, Spearman's rho ($r_s$). For all statistical tests used, $p < .05$ was considered significant. Data are presented as mean ± *SD*, unless otherwise stated.

### 3 | RESULTS

One patient dataset was excluded for excessive motion artifacts on MRI data. A summary of subject characteristics and clinical hematologic parameters of the included study subjects is provided in Table 2. All patient hematologic values were acquired during a routine clinic visit within 17 ± 13 days of the MRI scan. Blood measures for healthy controls were not collected. Eleven patients did not currently undergo treatment for SCD and one patient was receiving hydroxyurea therapy (16.7 mg $kg^{-1}day^{-1}$). Structural MRI data were reviewed for all subjects and five of 12 SCD patients presented with SCI. Within this group, 3/5 patients presented with bilateral SCI. No significant vessel stenosis or tortuosity was identified on MRA in either patients or controls.

In SCD patients, the average grey matter $CBF_{ASL}$ for the MCA territory was 61.0 ± 10.4 ml/min per 100 g. In controls, the average $CBF_{ASL}$ was 38.4 ± 7.0 ml/min per 100 g. A representative CBF map for a SCD patient is shown in Figure 2. Mean $CBF_{ASL}$ in SCD patients was significantly higher compared to control subjects ($p < .001$, Welch's *t* test). Results are summarized in Table 3, along with measured MCA diameter and weight of MCA territory used for conversion.

**TABLE 2** Subject characteristics (mean ± *SD*) and hematologic parameters (median ± median absolute deviation)

| Variable | SCD Patients | Controls |
|---|---|---|
| Age (years) | 15.1 ± 3.0 | 15.1 ± 2.4 |
| Gender (Male/Female) | 2/10 | 4/4 |
| Genotype | 12 HbSS | – |
| Stenosis | 0/12 | 0/8 |
| Silent infarction | 5/12 | 0/8 |
| $SpO_2$ (%) | 98.0 ± 3.0 | – |
| Hematocrit (%) | 26.0 ± 3.5 | – |
| Hemoglobin (g/L) | 91.0 ± 17.8 | – |
| Absolute Reticulocyte Count (K/$mm^3$) | 195 ± 64 | – |
| White Blood Cell Count (×$10^9$/L) | 9.2 ± 2.4 | – |
| Neutrophil Count (×$10^9$/L) | 5.05 ± 1.9 | – |
| Platelet Count (×$10^9$/L) | 398 ± 33 | – |
| Lactate Dehydrogenase (IU/L) | 990 ± 546 | – |

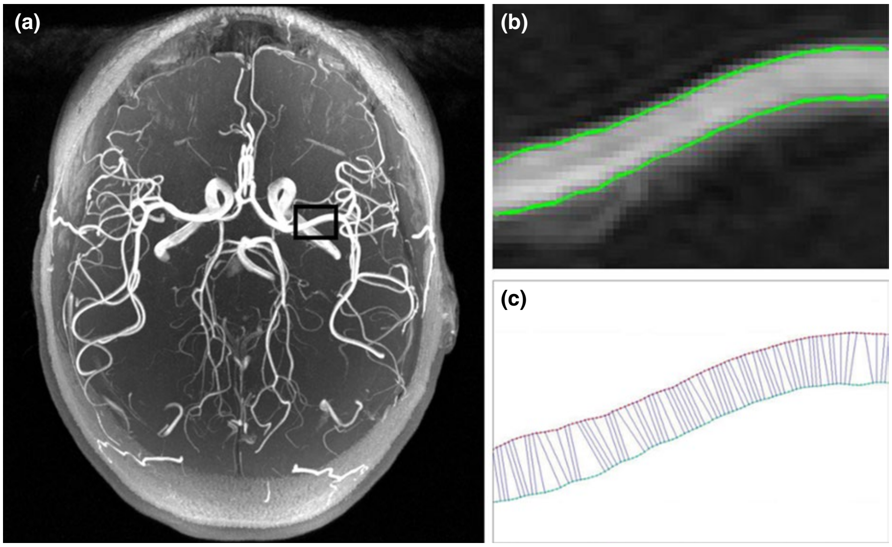

**FIGURE 1** Measurement of mean middle cerebral artery (MCA) diameter in a representative subject. (a) The M1 segment of the right MCA is isolated from a time-of-flight image (black outline). (b) The vessel walls are automatically traced using a contour algorithm. (c) The mean diameter of the vessel segment is calculated by averaging the minimum distance between the contours at multiple sample points

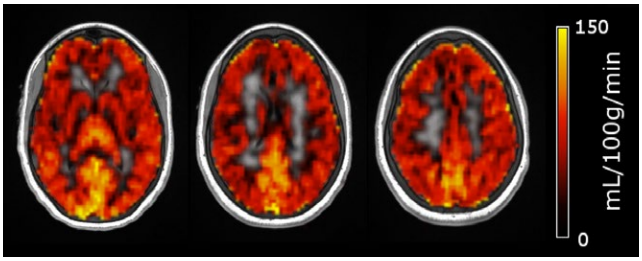

**FIGURE 2** Axial slices of a CBF map obtained using ASL in a representative SCD patient

The average $BFV_{MCA}$ was 93.6 ± 21.2 cm/s in SCD patients and 62.6 ± 17.8 cm/s in controls, demonstrating a significant elevation in the patient group ($p$ = .003, Welch's $t$-test). After conversion from $BFV_{MCA}$ into $CBF_{TCD}$, the group difference remained significant ($p$ < .001, Welch's $t$-test), with average $CBF_{TCD}$ of 66.5 ± 20.8 ml/min per 100 g in SCD patients and 31.0 ± 6.5 ml/min per 100 g in controls.

No significant association was observed between mean grey matter $CBF_{ASL}$ in the MCA territory and corresponding $BFV_{MCA}$ ($r$ = .275, $p$ = .389 in patients; $r$ = .103, $p$ = .808 in controls), as shown in Figure 3. However, after conversion from TCD measurements of $BFV_{MCA}$ into $CBF_{TCD}$, there was a significant correlation between $CBF_{ASL}$ and $CBF_{TCD}$ ($r$ = .671, $p$ = .017 in patients; $r$ = .862, $p$ = .006 in controls) (Figure 4). To illustrate the overall agreement, Bland–Altman plots have been included in Figure 5. For both patients and controls, the association between $CBF_{ASL}$ and $CBF_{TCD}$ remained significant after controlling for potential age effects.

Bilateral averages were subsequently compared to $CaO_2$ in patients. A strong association was observed between $CBF_{ASL}$ and $1/CaO_2$ ($r$ = .78, $p$ = .003), however, no significant association was observed with $BFV_{MCA}$ ($r$ = .22, $p$ = .491). After conversion from $BFV_{MCA}$ into $CBF_{TCD}$, a stronger association was observed between $CBF_{TCD}$ and $1/CaO_2$ ($r$ = .35, $p$ = .271), although it remained below statistical significance.

### 4 | DISCUSSION

We present a comparison between TCD measurements of MCA velocity and ASL measurements of CBF in the MCA territory in pediatric

**TABLE 3** Absolute hemodynamic and morphological measurements in SCD patients and healthy controls

| | SCD Patients | Controls | $p$-value |
|---|---|---|---|
| $CBF_{ASL}$ (ml/min/100 g) | 61.0 ± 10.4 | 38.4 ± 7.0 | <.001 |
| $BFV_{MCA}$ (cm/s) | 93.6 ± 21.2 | 62.6 ± 17.8 | .003 |
| MCA Diameter (mm) | 3.77 ± 0.56 | 3.45 ± 0.49 | .179 |
| MCA territory weight (g)[a] | 480 ± 23 | 575 ± 20 | .007 |
| $CBF_{TCD}$ (ml/min/100 g) | 66.5 ± 20.8 | 31.0 ± 6.5 | <.001 |

[a]Non-normally distributed data, displayed as median ± median absolute deviation.

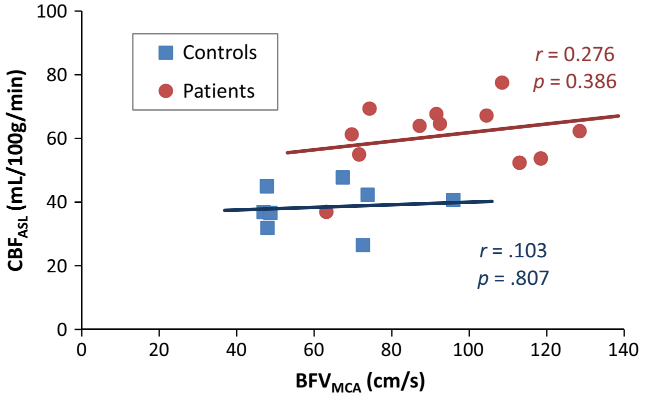

**FIGURE 3** Association between grey matter cerebral blood flow ($CBF_{ASL}$) in the middle cerebral artery (MCA) territory and corresponding MCA blood flow velocity ($BFV_{MCA}$). No significant association was observed in patients ($N$ = 12, $r$ = .28, $p$ = .39) or healthy controls (N = 8, $r$ = .10, $p$ = 0.81)

patients with SCD as well as healthy controls. First-order analysis showed no significant association between the two measurements, consistent across both children with SCD and age-matched controls. However, after conversion from TCD velocity measurements into CBF to account for vessel area and tissue weight, a significant correlation between the TCD and ASL measurements of CBF was observed.

Our findings suggest that the observed discrepancy between TCD measures of $BFV_{MCA}$ and ASL measures of tissue-level CBF in previous studies of SCD predominantly reflects the different physical principles which underlie these two measurements. CBF, or perfusion, is the volumetric flow of blood to an absolute mass of brain tissue, where volumetric flow is defined as the product of vessel cross-sectional area and velocity of moving blood, and tissue mass a function of density and volume. Therefore, the assumption that TCD measurements of BFV can be used as an accurate surrogate for CBF can only be true if both vessel area and tissue weight remain constant between individuals. It becomes even more important to correct for vessel diameter in SCD, as the diameter of the large intracranial arteries may become

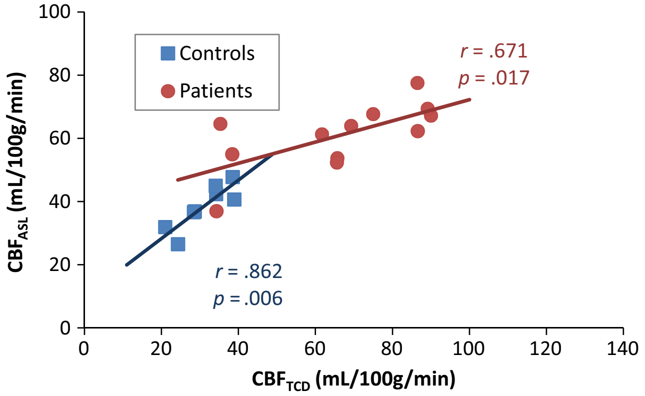

**FIGURE 4** Association between grey matter $CBF_{ASL}$ in the middle cerebral artery (MCA) territory and converted $CBF_{TCD}$. A significant linear association was observed in both patients ($N$ = 12, $r$ = .67, $p$ = .017) and healthy controls ($N$ = 8, $r$ = .86, $p$ = .006)

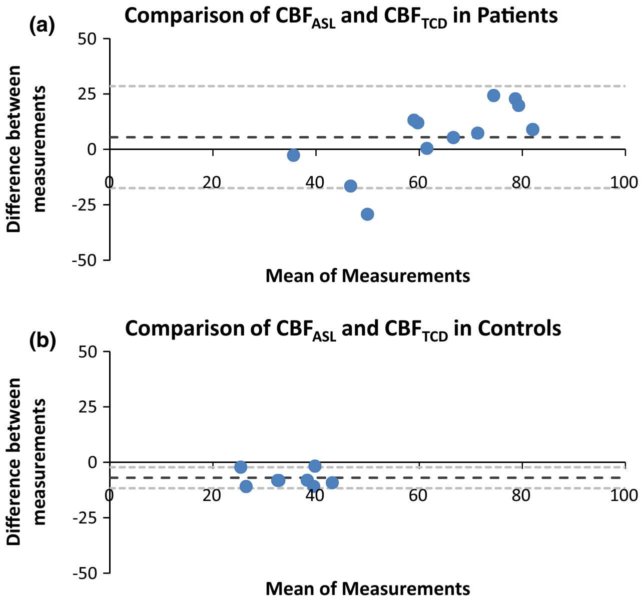

**FIGURE 5** Bland–Altman plots showing the agreement between $CBF_{ASL}$ and $CBF_{TCD}$ averaged within the middle cerebral artery (MCA) territories for (a) sickle cell disease (SCD) patients and (b) healthy controls dilated as an adaptive response to chronic anemia, or stenosed due to vasculopathy (Adams, Mckie, Brambilla, et al., 1998). However, it is worth noting that the strength of the correlation between the two CBF measures is stronger in the control group, in comparison to children with SCD, suggesting there may still be some contribution from SCD pathophysiology altering the vascular organization of the brain. For this study, the MCA perfusion territories were identified based on atlases defined within healthy individuals only (Moeller & Reif, 2013). The combined effect of chronic anemia and microvascular occlusions in SCD can affect the distribution of blood supply in the brain and could potentially lead to deviations in the overall size and geometry of the MCA territories. While there is currently no available data to confirm this, recent advances in ASL have enabled vessel selective encoding that can shed light into this issue. Helton et al. (2015) showed that accurate perfusion territory masks can be semiautomatically generated from vessel-encoded ASL in children with SCD, providing a means to develop population-based statistics. As the effect of age and disease on perfusion territories becomes better understood, it may be feasible to rely on literature values of MCA territory volume instead of measuring it with MRI.

TCD is a convenient clinical modality, as it is widely available, portable, and relatively easy to implement. While the ability of TCD to predict and prevent overt stroke in children with SCD (Adams, McKie, Hsu, et al., 1998) is clinically accepted, there is a recognized trade-off between sensitivity and specificity. While the clinical utility of TCD for predicting overt stroke risk is not under question, it has recently been proposed by Hulbert & Ford (2014) that we begin to move beyond global TCD measurements to regional MRI measurements to better understand tissue-level pathophysiology. Our findings suggest that global TCD values can be only used as a surrogate marker for tissue-level physiology, if measurements of diameter and density are available, which may undermine the potential of $CBF_{TCD}$ for tissue-level applications. Furthermore, our findings demonstrate that a strong inverse association exists between $CBF_{ASL}$ and $CaO_2$, which is a known predictor of ischemia (DeBaun, Sarnaik, et al., 2012; Quinn et al., 2009). Our data do not show a significant relationship between $BFV_{MCA}$ and $CaO_2$. Interestingly, after conversion into $CBF_{TCD}$, a stronger association is observed between $CBF_{TCD}$ and $CaO_2$, but the results were still not significant. We therefore propose that MRI measurements of tissue-level CBF, such as ASL, may be valuable in children with SCD for understanding more subtle pathophysiology such as SCI. It is worth noting that while we pose that tissue-level measures of CBF may be more sensitive to subtle ischemic damage, we have not tested the sensitivity of ASL to SCI. This is because we presented ASL measurements averaged across the entire MCA territory for the purposes of comparison to TCD measures. Averaging over such a large area obscures regional details necessary for identifying subtle changes caused by SCIs. CBF analysis based on much smaller regions, such as on a voxel-wise basis, could potentially detect the presence of SCIs, but this is outside the scope of our comparative study and will but explored in future research.

It has recently been recommended that surveillance MRI be performed in all children with SCD as a new standard of care to allow early detection of SCI and subsequent therapeutic intervention (Cancio et al., 2015; DeBaun et al., 2014). We recommend based on the findings of this study, that physiological measures, such as CBF quantification with ASL, be considered for inclusion to clinical MRI protocol in children with SCD. By only increasing the scan time by approximately 5 min, valuable regional insight into tissue-level cerebrovascular health can be gained. However, ASL is still an emergent technology and further validation and standardization (across populations, sequences, and vendors) are still necessary before widespread clinical implementation can be considered. Moreover, the clinical impact of local flow abnormalities in SCD has still not been fully established. An alternative strategy in the interim may be to include phase-contrast MRI to quantify bulk flow into the brain. A previous study has shown a strong correlation between $BFV_{MCA}$ measured using phase contrast and TCD (Leung, Behpour, Sokol, Mohanta, & Kassner, 2013). While this approach will not be able to detect local perfusion abnormalities, phase-contrast imaging is relatively fast and can measure vessel caliber more reliably than TCD.

Our overall sample size was limited because it was based on available data from an earlier study. Thus, bias may arise from the lack of gender matching between patients and controls, which is unlikely to impact within-group comparison but may contribute to between-group differences. The current findings should be considered preliminary and can benefit from a replication study with a larger sample. Moreover, we did not have blood test results from our healthy control group, which means the Hct correction for our CBF measures is based on assumed values. However, the influence of Hct on CBF quantification is typically small for subjects with normal blood parameters. Only in cases of anemia like in SCD will the impact of Hct correction have a significant impact. Another limitation of this study is that our measured

$BFV_{MCA}$ and CBF values are potentially underestimated for both patients and controls in comparison to literature values (Gevers et al., 2012; Herold et al., 1986; Petersen, Lim, & Golay, 2006; Prohovnik et al., 2009; Schöning, Staab, Walter, & Niemann, 1993; Strouse et al., 2006), which may be attributed to both technical and/or physiological factors.

For TCD measurements, underestimation may arise from our choice of TCD technique. TCD imaging, as used in this study, has been reported to underestimate $BFV_{MCA}$ by approximately 9% in comparison to nonimaging TCD methods (Bulas et al., 2000). In addition to TCD method choice, there may be additional contributions in some subjects due to an incorrect angle of insonation. We assumed the angle to be less than 10° for all measurements, but if the actual angle was greater, $BFV_{MCA}$ may be underestimated (Leung et al., 2013). Angle correction was not performed, however, in order to conform to standard clinical protocol. In addition, it is possible that systematic error in the conversion into $CBF_{TCD}$ can be introduced from the semiautomated estimation of the MCA diameter. The vessel diameter is computed from time-of-flight images using thresholding and contouring algorithms, which are dependent on the analysis parameters. While we used consistent parameters across all subjects, the validation of our measurements was not feasible as it would likely require highly invasive procedures.

For ASL measurements, MRI sequence parameters were chosen based on a clinically approved protocol, which may not be optimal for the high-flow environments observed in both pediatric subjects and SCD patients, leading to values lower than expected for both groups. While Pulsed ASL is in theory robust to fluctuations in velocity (Wong, Buxton, & Frank, 1998b), it remains plausible that this has contributed to an inversion efficiency lower than the assumed 95%, which would lead to a systematic underestimation of CBF. Additionally, ASL data were quantified according to the single-compartment model; in instances of short arterial transit time, this may lead to an underestimation of CBF due to incorrect modeling of $T_1$ effects. While a two-compartment model may help resolve this issue (Parkes, 2005), bias would likely still arise from errors in the outflow term, with additional error propagated from the need to assume venous magnetization. Furthermore, for both techniques, discrepancies between our measured values and previous literature may arise from differences in subject demographics such as age (Leung, Kosinski, Croal, & Kassner, 2016), and in the case of SCD patients, severity of anemia (Prohovnik et al., 1989). However, such factors do not impede the ability to assess relative differences between groups.

While this study seeks to compare TCD and ASL measurements of blood flow abnormalities in children with SCD, we did not assess their relative potential to predict ischemic events. Thus, the clinical value of tissue-level perfusion over flow velocity in sickle cell disease is yet to be established. However, we have shown that ASL measures of blood flows are significantly correlated with hematologic predictors of ischemia, in agreement with recent findings (Bush et al., 2016; Kosinski et al., 2017).

The current results warrant further investigation into the role of ASL in predicting silent and overt ischemia as TCD measures of $BFV_{MCA}$ appear to be too distal to the site of injury to be effective. In particular, the inclusion of longitudinal data in future studies, including patients with a history of neurologic injury, would allow us to assess whether ASL measurements of tissue-level CBF are able to predict primary or recurrent ischemic events. There are limited ASL studies in SCD to date (Gevers et al., 2012; van der Land et al., 2015; Oguz et al., 2003; van den Tweel et al., 2009), however, none as of yet have fully addressed the technical challenge of accurate perfusion quantification under the high-flow environment in SCD.

Our findings show a lack of association between TCD measures of $BFV_{MCA}$ and ASL MRI measures of CBF, consistent across both children with SCD and healthy age-matched controls. However, a strong positive association exists if individual differences in vessel diameter and tissue weight are taken into account. Furthermore, such tissue-level measurements show an improved association with hematologic risk factors for ischemia. We highlight the potential value of including MRI measurements of tissue-level perfusion if already performing surveillance with anatomical MRI, in order to understand and predict the more subtle neurological effects of SCD.

### ACKNOWLEDGMENTS AND DISCLOSURE

This work was supported by the Canadian Institutes of Health Research [grant number 1111113] and Canada Research Chairs [grant number 950-220786]. The funding sources had no involvement in the study design; in the collection, analysis, and interpretation of data; in the writing of the report; and in the decision to submit the article for publication.

### ORCID

*Andrea Kassner* http://orcid.org/0000-0002-1071-5101
